# Genome insights into the plant growth-promoting bacterium *Saccharibacillus brassicae* ATSA2^T^

**DOI:** 10.1186/s13568-023-01514-1

**Published:** 2023-01-21

**Authors:** Lingmin Jiang, Jiyoon Seo, Yuxin Peng, Doeun Jeon, Soon Ju Park, Cha Young Kim, Pyoung Il Kim, Chul Hong Kim, Ju Huck Lee, Jiyoung Lee

**Affiliations:** 1 Biological Resource Center, Korea Research Institute of Bioscience and Biotechnology, Jeongeup, 56212 Republic of Korea; 2 Division of Applied Life Science, Gyeongsang National University, Jinju, 52828 Republic of Korea; 3 Center for Industrialization of Agricultural and Livestock Microorganisms (CIALM), Jeongeup, 56212 Republic of Korea; 4 Hyungnong Co., Ltd., Gokseong, 57512 Republic of Korea

**Keywords:** Endophyte, *Saccharibacillus brassicae*, Plant growth-promotion, Whole-genome, antiSMASH

## Abstract

Endophytes can facilitate the improvement of plant growth and health in agriculturally important crops, yet their genomes and secondary metabolites remain largely unexplored. We previously isolated *Saccharibacillus brassicae* strain ATSA2^T^ from surface-sterilized seeds of kimchi cabbage and represented a novel species of the genus *Saccharibacillus*. In this study, we evaluated the plant growth-promoting (PGP) effect of strain ATSA2^T^ in kimchi cabbage, bok choy, and pepper plants grown in soils. We found a significant effect on the shoot and root biomass, and chlorophyll contents following strain ATSA2^T^ treatment. Strain ATSA2^T^ displayed PGP traits such as indole acetic acid (IAA, 62.9 μg/mL) and siderophore production, and phosphate solubilization activity. Furthermore, genome analysis of this strain suggested the presence of gene clusters involved in iron acquisition (*fhuABD*, *afuABC*, *fbpABC*, and *fepCDG*) and phosphate solubilization (*pstABCHS*, *phoABHLU*, and *phnCDEP*) and other phytohormone biosynthesis genes, including indole-3-acetic acid (*trpABCDEFG*), in the genome. Interestingly, the secondary metabolites cerecidin, carotenoid, siderophore (staphylobactin), and bacillaene underlying plant growth promotion were found in the whole genome via antiSMASH analysis. Overall, physiological testing and genome analysis data provide comprehensive insights into plant growth-promoting mechanisms, suggesting the relevance of strain ATSA2^T^ in agricultural biotechnology.

**Supplementary Information:**

The online version contains supplementary material available at 10.1186/s13568-023-01514-1.

## Introduction

Plant-associated bacteria ubiquitously inhabit the rhizosphere (soil-root interface), phyllosphere (aerial surfaces), and endosphere (inside of the plant tissues). These bacteria may contribute to plant growth and health by assisting in nutrient uptake or by suppressing plant pathogens (Vejan et al. 2016; Afzal et al. 2019; Saberi Riseh et al. 2021; Majeed et al. 2018; Bhardwaj et al. 2014; Nanjani et al. 2022). Many studies have demonstrated plant–microbe symbiosis, in which bacteria produce extracellular enzymes or metabolites, thereby improving mineral nutrition and thus impacting plant health and growth, while plants provide energy to those beneficial microbes (Marschner and Rengel 2007; Santoyo et al. 2016; Hardoim et al. 2008; Zhao et al. 2022; Thiruvengadam et al. 2022). Rhizospheric and endophytic bacteria, which are cost-effective and environmentally friendly, have been widely used in biological control methods and plant biofertilizers. For example, many *Bacillus* isolates have been shown to have antifungal activity against phytopathogenic fungi, making them good biocontrol candidates (Chen et al. 2019; Zaid et al. 2022). In addition, *Enterobacter roggenkampii* and *Pseudomonas aeruginosa* have been used to promote sugarcane growth, while the *Burkholderia seminalis* strain promotes the growth of some vegetables and crops (Guo et al. 2020; Chandra et al. 2021; Singh et al. 2021; Hwang et al. 2021). Furthermore, *Pantoea*, *Pseudomonas*, *Burkholderia*, *Stenotrophomonas*, *Micrococcus*, *Streptomyces*, and *Microbacterium* are the most frequently used endophytic bacteria isolated from plants (Vejan et al. 2016; Afzal et al. 2019; Saberi Riseh et al. 2021; Majeed et al. 2018; Bhardwaj et al. 2014). The use of endophytic bacteria in agriculture has received increased interest in recent years for its potential to sustainably improve plant production (Singh and Gupta 2015; Vejan et al. 2016; Afzal et al. 2019; Majeed et al. 2018; Wang et al. 2022).

The mechanisms behind plant growth, both direct and indirect, have been extensively studied (Santoyo et al. 2016). Indirect mechanisms promote plant growth through the inhibition of phytopathogens, for example, via inhibition of soil-borne fungi, bacteria, and nematodes through the production of antimicrobial metabolites or siderophores (Fu et al. 2016; Singh et al. 2021); via the reduction of abiotic drought or salt stress (Saberi Riseh et al. 2021; Bhardwaj et al. 2014; Majeed et al. 2018; Vurukonda et al. 2016); or via the production of extracellular enzymes, including cellulase, protease, amylase, and chitinase (Vejan et al. 2016; Afzal et al. 2019; Saberi Riseh et al. 2021; Majeed et al. 2018; Bhardwaj et al. 2014). These enzymes play an important role in biodegradation, hydrolysis processes and induced systemic resistance, which decreases the iron content of pathogens and facilitates plant growth. For example, proteases, which are cell wall-degrading enzymes, suppress the growth of fungal pathogens and compete with deleterious fungal pathogens for nutrients to indirectly stimulate plant growth (Fu et al. 2016). Direct mechanisms promote plant growth by producing of phytohormones, such as auxins indole-3-acetic acid (IAA) (Spaepen et al. 2007; Fu et al. 2016), cytokinins or gibberellins (Sakakibara 2006; Zaidi et al. 2015), ACC deaminase (Vurukonda et al. 2016), and resource acquisition by nitrogen fixation (Geddes et al. 2015), phosphate solubilization (Singh and Gupta 2015), siderophores (Olanrewaju et al. 2017), and iron uptake (Kielak et al. 2016). Despite many excellent reviews summarizing working models of plant growth-promoting bacteria, the actual mechanisms of these effective bacteria remain unclear and require further exploration.

Genome sequencing is now an important component of natural product research. Whole-genome sequencing (WGS) enables the identification of the genes responsible for the biosynthesis of natural products (Scherlach and Hertweck 2021; Albarano et al. 2020; Baltz 2019; Bauman et al. 2021; Rikame and Borde 2022; Belaouni et al. 2022). Often, genes required for the biosynthesis of a natural product are positionally clustered on the genome and are referred to as biosynthetic gene clusters (BGCs) (Scherlach and Hertweck 2021; Albarano et al. 2020; Baltz 2019; Bauman et al. 2021). The BGC sequences can be used to predict possible structures of the resulting natural product (Albarano et al. 2020; Baltz 2019; Bauman et al. 2021), assess novel compounds and dereplicate compounds from a strain collection (Guo et al. 2020; Chandra et al. 2021; Singh et al. 2021; Hwang et al. 2021). However, to date, most studies have not used whole-genome sequencing to discover the enormous potential of endophytic bacteria or fungi for secondary metabolite production. Secondary metabolites are low-molecular-mass organic compounds that, unlike primary metabolites, are not directly involved in the growth, development or reproduction of the producing organism. The majority of bacterial secondary metabolites are derived from either nonribosomal peptide synthetase (NRPS) or polyketide synthase (PKS). The unknown metabolic pathways are likely to encode numerous bioactive molecules. One of the most commonly used tools, antiSMASH (Blin et al. 2019), has had a major impact on genome-to-genome correlation, query and prediction of natural product synthetic gene clusters.

*Saccharibacillus* is a genus of gram-positive, endospore-forming, facultatively anaerobic rod-shaped bacteria, of which there are seven published species, with the major species having been isolated from plants (Jiang et al. 2020; Darji et al. 2021; Kampfer et al. 2016; Rivas et al. 2008). To date, there have been no functional studies in plants with bacteria of this genus, and few secondary metabolites have been studied. The potential source of natural products in this genus remains unclear. We isolated an endophytic bacterium, ATSA2^T^, from kimchi cabbage seeds and later identified it as *Saccharibacillus brassicae* (Jiang et al. 2020)*.* Kimchi cabbage is an important vegetable in both Chinese and Korean cuisine. Kimchi cabbage is also a common crop in Europe, the Americas, and Australia due to its high nutritional value and its anticancer, antioxidative, and antiaging properties. The use of the microbiome may also be useful for improving cabbage agricultural production. In the present study, we verified the plant growth-promoting effect of ATSA2^T^ on kimchi cabbage, bok choy, and pepper grown in soils. Subsequently, the plant growth promotion (PGP)-associated characteristics of this strain were further identified from whole-genome analysis. We aimed to study strain ATSA2^T^ endophyte information, including genes related to PGP, extracellular enzyme-related genes, and metabolic profiling of the whole genome. The present study shows that genomic analysis offers comprehensive insights into the mechanisms of PGP, suggesting the relevance of strain ATSA2^T^ in agricultural biotechnology.

## Materials and methods

### Bacterial cultures

Strain ATSA2^T^ was isolated from surface-sterilized kimchi cabbage (*Brassica rapa* subsp. *pekinensis*) seeds and identified as *S. brassicae* in a previous study (Jiang et al. 2020). Unless otherwise stated, this strain was grown in tryptic soy broth (TSB, Difco) at 25 °C for 2 days until cultures reached the exponential growth phase.

### Plant growth promotion assay in plants

Various plant species, including kimchi cabbage, bok choy, and pepper, were subjected to bacterial inoculation assays (Hwang et al. 2021). Seeds were surface sterilized (Jiang et al. 2019a) and grown in autoclaved soils (121 °C for 15 min) at 25 °C (16 h light/8 h dark) in a seedling tray for kimchi cabbage and bok choy which were obtained from Asia seed Co., Ltd (Seoul, Korea), and pepper seeds from Nongwoo Bio Co., Ltd (Suwon, Korea). Bacterial treatments started when the plantlets had fully expanded cotyledons and the first true leaf was visible (5 days for bok choy and kimchi cabbage, and 7 days for pepper after sowing). Cells of strain ATSA2^T^ were grown in TSB at 25 °C for 2 days, and the bacterial suspension was then adjusted to 1 × 10^7^ cfu mL^−1^ (OD_600_ = 0.1). Next, 5 mL of bacterial suspension was poured into the potting soils of each plant seedling. Following inoculation, seedlings continued to grow in the greenhouse until the leaves fully expand in the pot for 21 days for bok choy and kimchi cabbage and 28 days for pepper. After co-cultivation with the bacterial strain, the weight of the roots after careful removal of soils, the number of leaves, and the leaf biomass were measured. All experiments were performed in duplicate with similar results.

Leaves were placed in a sterile 50 mL tube containing 15 mL of ethanol and then stored in the dark until the leaves turned white. Following extraction, chlorophyll A and B contents were measured at an absorbance of 663 nm and 645 nm, respectively, using a microplate reader (ThermoFisher Scientific, Multiskan SkyHigh). Total chlorophyll content (per plant) was calculated according to the following equation: *y* = 8.02 × A_663_ + 20.2 × A_645_.

### In vitro assessment of PGP traits

#### Quantitative evaluation of hydrolytic activities

Extracellular enzymatic activities of strain ATSA2^T^, including amylase, cellulase, protease, and xylanase, were performed as previously described (Fu et al. 2016; Fouda et al. 2015; Maheshwari et al. 2020). Briefly, 5 μL of bacterial suspension (OD_600_ = 0.1) was spotted onto tryptic soy agar (TSA) supplemented with specific indicative substrates and incubated at 25 °C for 5 days. Amylase and cellulase activities were determined by spotting samples on TSA containing 1% (w/v) soluble starch and 1% (w/v) carboxymethylcellulose (CMC). Next, plates were flooded with 1% (w/v) iodine, and the appearance of clear zones around colonies was measured. Protease activity was determined using TSA medium supplemented with 1% (w/v) gelatin, and any clear zones around colonies were measured after using 4.1 M (NH_4_)_2_SO_4_ solution. Xylanase activity was assessed using TSA medium supplemented with 1% (w/v) xylan from corncobs. Clear zones were then visualized with 0.1% (w/v) Congo red. All experiments were performed in triplicate. The clear zone and the colony diameter of each repeat were measured using ImageJ software (n = 9).

#### IAA production

Indole acetic acid (IAA) production was performed as previously described (Hwang et al. 2021) with minor modifications. Briefly, 100 μL of bacterial suspension [optical density at 600 nm (OD_600_) = 0.1] was grown in 10 mL of TSB with or without 0.1% (w/v) tryptophan (L-Trp) with agitation at 150 rpm for 5 days in the dark. Next, 1 mL of each culture was collected into 1.5-mL Eppendorf tubes and centrifuged at 8000×*g* for 10 min at room temperature. Subsequently, 500 μL of the supernatant was mixed with an equal volume of Salkowski’s reagent (2 mL of 0.5 M FeCl_3_; 98 mL H_2_SO_4_). After 30 min of incubation at room temperature, the concentration of IAA was quantified colorimetrically by measuring the optical density at 530 nm (Asghar et al. 2002) using a spectrophotometer (Optizen POP). Bacterial IAA production was estimated against a standard curve (Ahmad et al. 2005) using commercial IAA (Sigma-Aldrich) at different concentrations (0, 10, 20, 30, 40, 50, and 60 μg/mL). This experiment was performed in independent triplicates.

#### Phosphate solubilization

Phosphate solubilization was assessed by placing 5 μL of bacterial suspension (OD_600_ = 0.1) on the National Botanical Research Institute's phosphate growth medium (NBRIP), which contained glucose (10 g/L), Ca_3_(PO_4_)_2_ (5 g/L), MgCl_2_-6H_2_O (5 g/L), MgSO_4_·7H_2_O (0.25 g/L), KCl (0.2 g/L), (NH4)_2_SO_4_ (0.1 g/L), and agar (15 g/L), and incubating at 25 °C for 5 days. Phosphate solubilizing index (PSI) was calculated as the ratio of (halo + colony)/colony diameters.

#### Siderophore production

Siderophore production was performed on chrome azurol S (CAS) agar using the diffusion assay method (Shin et al. 2001). The diameters of colonies and orange color zones surrounding the colonies were measured after 14 days in triplicate. The development of a yellow or orange halo around the colony was measured and calculated using the halo diameter to colony diameter ratio. Siderophore producing index (SPI) was calculated as the ratio of (colored zone + colony)/colony diameters.

#### Nitrogen fixation

The nitrogen fixation activity was detected on nitrogen-free Jensen’s medium, and isolates that continued to grow were further streaked onto Jensen’s media to confirm the nitrogen-fixing ability (Atencio et al. 2020).

### Genome mining

The whole genome of strain ATSA2^T^ was sequenced as described in our previous studies (Jiang et al. 2019b; Jiang et al. 2020) (accession number CP041217). The Kyoto Encyclopedia of Genes and Genomes (KEGG) GENOME database, RastSEED (Henry et al. 2010) and BlastKOALA were used for metabolic pathway analysis (Du et al. 2014; Kanehisa and Goto 2000; Kanehisa et al. 2016) and gene functional mining for PGP traits such as the phytohormone IAA. In addition, some useful secondary metabolite biosynthesis gene clusters and gene functions were predicted and classified using antiSMASH (v.5.1.1) (Blin et al. 2019) and the cluster of orthologous groups (COG) database (Tatusov et al. 2003). VirulenceFinder and ResFinder (https://cge.cbs.dtu.dk/services/) were used to identify genes involved in mycotoxin synthesis, bacterial pathogenicity toward human hosts, virulence, and antibiotic resistance (Kleinheinz et al. 2014). Parameters in VirulenceFinder and ResFinder were set to 60% minimum length threshold and 90% identity threshold.

### Statistical analysis

For each of the investigated plant biomass parameters from control and strain ATSA2^T^ treated plants, two separate replication sets were conducted. All the experimental measurement values were expressed as means of five to fifteen plants [± standard deviation (SD)]. The significance of the differences between the mean values of control and treated plants was significantly evaluated by unpaired *t*-test at P ≤ 0.05 using Prism’s GraphPad (Vers.8.2.1).

## Results

### Effects of *Saccharibacillus brassicae* strain ATSA2^T^ on plant growth promotion

As strain ATSA2^T^ was an endophytic bacterium isolated from seeds of kimchi cabbage, the functional roles were tested on rice and Micro-Tom tomato seed germination on filter paper and on plant growth on 1/2 MSBM medium (Additional file 1: Fig. S1). The results showed that coating seeds of rice and Micro-Tom with strain ATSA2^T^had relatively higher seed germination rates than the controls. The germination rate increased from 66 to 78% for rice and from 36 to 64% for Micro-Tom. Similar plant growth-promoting results were observed following co-culture on 1/2 MSBM medium after 7 days for rice and 14 days for Micro-Tom (Additional file 1: Fig. S1). These results confirm a potential role for ATSA2^T^ in promoting plant growth.

**Fig. 1:**
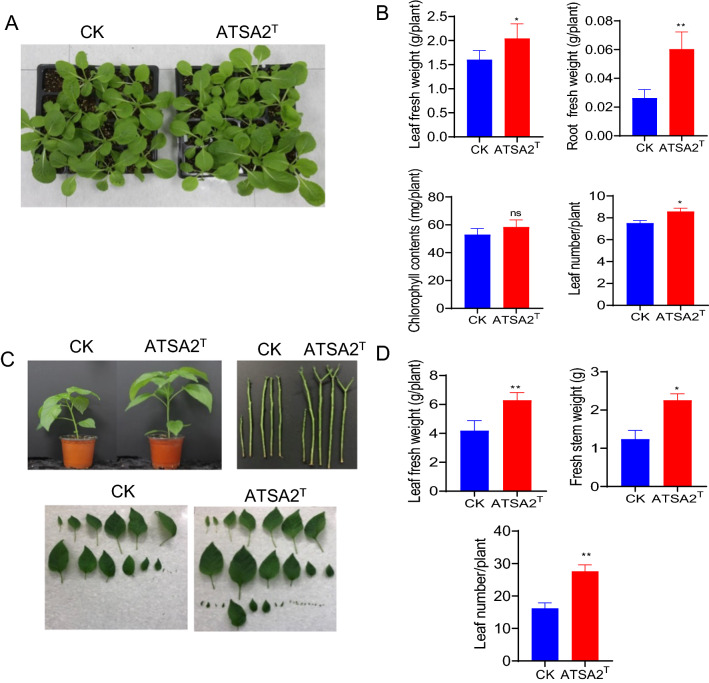
Effect of the strain ATSA2^T^ on kimchi cabbage and pepper plant growth. The growth of kimchi cabbage (**A**, **B**) and pepper (**C**, **D**) with and without ATSA2^T^ inoculation were determined at 21 and 28 days after inoculation, respectively. **A**, **C** Representative photograph showing the effects of the strain ATSA2^T^. **B**, **D** The average leaf fresh weight, root fresh weight, chlorophyll content, and leaf number of plants inoculated with ATSA2^T^. Error bars indicate the standard deviation of the mean (n = 5–15). Asterisks (*) indicate statistically significant differences between control (CK) and ATSA2^T^ inoculation (*P < 0.05, *t*-test). Experiments were repeated twice with similar results

To better apply the strain in the field, the PGP capacity of strain ATSA2^T^ was next tested in vegetable plants, including the plant genus *Brassica* as kimchi cabbage and bok choy, and genus *Capsicum* as pepper (*Capsicum annuum* L.), grown on soils, as these vegetables are the most popular foods in Korea family known as Kimchi. After cocultivation with strain ATSA2^T^ for 21 days for kimchi cabbage and bok choy, and 28 days for pepper plants, the bacteria-treated plants were visually longer and had larger leaves than the controls (Fig. 1A, C and Additional file 1: Fig. S2A). Following the assessment of biometric parameters, the leaf fresh weight, root fresh weight, chlorophyll content, and leaf number of kimchi cabbage cocultured with strain ATSA2^T^ were increased by 1.12-fold, 2.15-fold, 1.06-fold, and 1.14-fold, respectively, compared to control (Fig. 1B). Similar observations were made for bok choy plants, in which the leaf fresh weight, root fresh weight, and leaf number increased by 1.32-fold, 2.27-fold, and 1.10-fold, respectively (Additional file 1: Fig. S2B). These data were further supported by the results from the pepper plants, which showed 1.50-fold, 1.83-fold, and 1.70-fold increased leaf fresh weight, stem weight, and total leaf number, respectively, following coculture with strain ATSA2^T^ (Fig. 1D). Collectively, these data suggest that strain ATSA2^T^ has plant growth-promoting activity against kimchi cabbage, bok choy, and pepper.

### PGP trait assessment in vitro

Strain ATSA2^T^ was found to produce 62.9 μg/mL IAA when cultured in TSB with Trp as the biochemical precursor (Table 1) and 6.1 µg/mL IAA without Trp. Strain ATSA2^T^ did not show extracellular enzymatic activity such as amylase, cellulase, protease, and xylanase, but showed positive siderophore production and phosphate solubilization activity (Table 1).

**Table 1:** Auxin (IAA) and siderophore production, P-solubilization, and extracellular enzyme activities of strain ATSA2^T^

Plant growth-promoting trait	ATSA2^T^
IAA production	62.9 µg/mL
Siderophore production index (SPI)^a^	1.43 ± 0.02
P-solubilization index (PSI)^a^	1.18 ± 0.02
N-fixation	–
Amylase	–
Cellulase	–
Xylanase	–
Protease	–

–: not identified

^a^Data are presented as means ± standard errors of triplicates (n = 9)

### Genome characteristics of strain ATSA2^T^

**Fig. 2:**
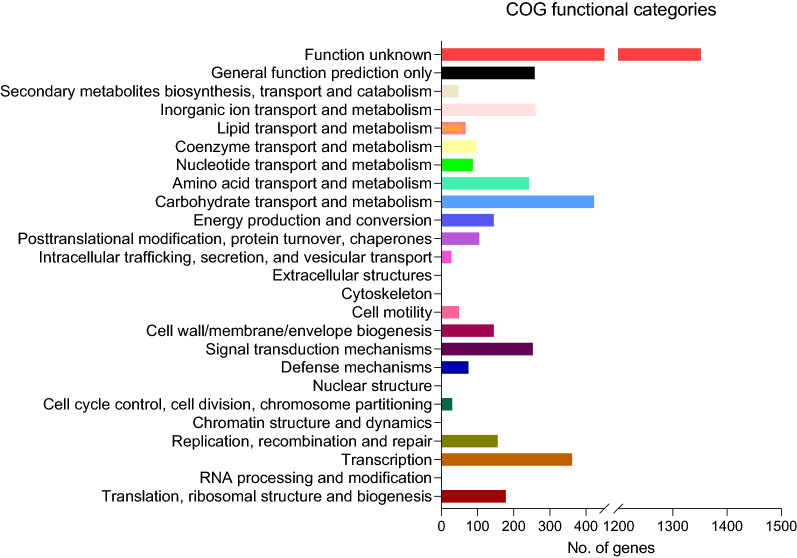
The cluster of orthologous groups (COG) classification of putative proteins in the ATSA2^T^ genome

The whole-genome sequence of strain ATSA2^T^ features one 5,619,468-bp circular chromosome. COG annotation results showed 4604 protein-coding genes (CDSs) and 4312 eggnog database-matched proteins in the whole genome. Interestingly, 1.7% (75) of the genes corresponded to defense mechanisms, 1.1% (49) were associated with motility, 3.3% (145) of the genes were related to the cell wall and membrane biosynthesis, 5.6% (243) of the genes corresponded to amino acid transport and metabolism, and 6.0% (260) of the genes were grouped into ion transport and metabolism (Fig. 2). These classes are implicated in bacteria–host communication mechanisms (Turkina and Vikstrom 2019). Bacteria can recognize plant-derived molecules to communicate with their host plant via the biosynthesis of bacteria-derived molecules. For example, plants secrete rich biomolecules such as flavonoids, which attack bacteria, and bacteria control their swarming activity to live on the plant surfaces or even inside to form architecturally complex biofilms, producing abundant plant growth substances, promoting plant growth and protecting against pathogens (Guo et al. 2015). Further analysis is required to understand the characteristics of these unique genes.

The whole genome of strain ATSA2^T^ was analyzed for virulence and antibiotic resistance genes using a user-friendly web tool provided by the Center for Genomic Epidemiology (CGE). The results showed that the ATSA2^T^ genome did not contain any antibiotic resistance genes. This strain is sensitive to all antibiotics, including but not limited to chloramphenicol, kanamycin, nalidixic acid, nitrofurantoin, penicillin, and tetracycline, which is consistent with previous studies (Jiang et al. 2020). Moreover, we did not detect any virulence genes. These results suggest that strain ATSA2^T^ could be used as a safe biofertilizer for field application.

### Plant growth-promoting traits from the whole genome

The ATSA2^T^ genome was analyzed to identify genes that are involved in PGP-related pathways or traits involving extracellular enzymes, including cellulase, chitinase, amylase and the phytohormone indole-3-acetic acid (IAA). We also sought to identify genes involved in iron-siderophore transport or important metabolic pathways, including ammonia assimilation, sulfate, and nitrogen metabolism, as well as phosphate solubilization.

#### IAA biosynthesis

In-vitro IAA production analysis showed that ATSA2^T^ could produce 62.9 µg/mL of IAA with Trp as a precursor. IAA, the most biologically active auxin, regulates plant growth and development by promoting cell proliferation and antioxidant effects. Furthermore, IAA is an important microbial tryptophan metabolite. We identified many genes implicated in IAA production and tryptophan synthase genes, such as *trpABCDEFG* (Table 2). In addition, *ipdC*, a key gene involved in the IAA biosynthesis pathway identified in *Klebsiella* sp. D5A (Liu et al. 2016), and *patB*, a gene that is potentially involved in an IAA biosynthetic pathway in *Bacillus amyloliquefaciens* Ba13, were also identified in the ATSA2^T^ genome (Ji et al. 2021) (Table 2).

**Table 2:** Genes involved in plant growth-promoting traits based on BlastKOALA and RastSEED

Traits	Gene	Gene annotation	E.C. Number
IAA	*trpD*	Anthranilate phosphoribosyltransferase	[EC:2.4.2.18]
	*trpC*	Indole-3-glycerol phosphate synthase	[EC:4.1.1.48]
	*trpA*	Tryptophan synthase alpha chain	[EC:4.2.1.20]
	*trpB*	Tryptophan synthase beta chain	[EC:4.2.1.20]
	*trpF*	Phosphoribosylanthranilate isomerase	[EC:5.3.1.24]
	*trpEG*	Anthranilate synthase	[EC:4.1.3.27]
	*ipdC*	Indolepyruvate decarboxylase	[EC:4.1.1.74]
	*WARS*, *trpS*	Tryptophanyl-tRNA synthetase	[EC:6.1.1.2]
	*mtrB*	Transcription attenuation protein (tryptophan RNA-binding attenuator protein)	
	*ipdC*	Indolepyruvate decarboxylase	[EC:4.1.1.74]
	*amiE*	Amidase	[EC:3.5.1.4]
	*patB*, *malY*	Cysteine-*S*-conjugate beta-lyase	[EC:4.4.1.13]
	*ND*	Putative tryptophan/tyrosine transport system substrate-binding protein	
	*ND*	Putative tryptophan/tyrosine transport system permease protein	
	*ND*	Putative tryptophan/tyrosine transport system ATP-binding protein	
Iron-siderophore transport	*sirC*, *fecD*, *cbrC*	Iron-siderophore transport system permease protein	
	*sirB*, *fecC*, *cbrB*	Iron-siderophore transport system permease protein	
	*sirA*, *fecB*, *cbrA*	Iron-siderophore transport system substrate-binding protein	
	*feoB*	Ferrous iron transport protein B	
	*ND*	Iron complex transport system substrate-binding protein	
	*fagD*, *cchF*, *irp1A*, *piaA*	Iron-siderophore transport system substrate-binding protein	
	*fepD*, *fagA*, *cchC*, *desH*	Iron-siderophore transport system permease protein	
	*efeO*	Iron uptake system component EfeO	
	*fepG*, *fagB*, *cchD*, *desG*	Iron-siderophore transport system permease protein	
	*fepC*, *fagC*, *cchE*, *desF*	Iron-siderophore transport system ATP-binding protein	[EC:7.2.2.17 7.2.2.-]
	*fhuB*, *ftsC*, *siuB*	Ferric hydroxamate/heme transport system permease protein	
	*fhuD*, *ftsB*, *siuD*	Ferric hydroxamate/heme transport system substrate-binding protein	
	*fhuA*, *ftsA*, *siuA*	Ferric hydroxamate/heme transport system ATP-binding protein	[EC:7.2.2.16]
Iron(III) transport	*afuB*, *fbpB*	Iron(III) transport system permease protein	
	*afuC*, *fbpC*	Iron(III) transport system ATP-binding protein	[EC:7.2.2.7]
	*afuA*, *fbpA*	Iron(III) transport system substrate-binding protein	
	*afuC*, *fbpC*	Iron(III) transport system ATP-binding protein	[EC:7.2.2.7]
	*afuA*, *fbpA*	Iron(III) transport system substrate-binding protein	
	*afuB*, *fbpB*	Iron(III) transport system permease protein	
	*ND*	Iron complex transport system ATP-binding protein	[EC:7.2.2.-]
Phosphate solubilization	*phnD*	Phosphonate transport system substrate-binding protein	
	*phnC*	Phosphonate transport system ATP-binding protein	[EC:7.3.2.2]
	*phnE*	Phosphonate transport system permease protein	
	*phnP*	Phosphoribosyl 1,2-cyclic phosphate phosphodiesterase	[EC:3.1.4.55]
	*phoA*, *phoB*	Alkaline phosphatase	[EC:3.1.3.1]
	*phoH*, *phoL*	Phosphate starvation-inducible protein *PhoH* and related proteins	
	*phoH2*	PhoH-like ATPase	
	*phoU*	Phosphate transport system protein	
	*pstA*	Phosphate transport system permease protein	
	*pstB*	Phosphate transport system ATP-binding protein	[EC:7.3.2.1]
	*pstC*	Phosphate transport system permease protein	
	*pstS*	Phosphate transport system substrate-binding protein	
	*ptsH*	Phosphocarrier protein HPr	
	*ugpA*	Sn-glycerol 3-phosphate transport system permease protein	
	*ugpB*	Sn-glycerol 3-phosphate transport system substrate-binding protein	
	*ugpE*	Sn-glycerol 3-phosphate transport system permease protein	
	*plsX*	Phosphate acyltransferase	[EC:2.3.1.274]
	*ppk1*	Polyphosphate kinase	[EC:2.7.4.1]
	*gapA*	Glyceraldehyde 3-phosphate dehydrogenase	[EC:1.2.1.12]
	*hisC*	Histidinol-phosphate aminotransferase	[EC:2.6.1.9]
	*TC.PIT*	Inorganic phosphate transporter, PiT family	
	*ugpE*	Sn-glycerol 3-phosphate transport system permease protein	

##### Cellulase

Although in-vitro cellulase production failed to highlight any cellulase activity in strain ATSA2^T^, some *Saccharibacillus* sp. strains showed significant amount cellulase (Rivas et al. 2008; Darji et al. 2021; Besaury and Remond 2020). Cellulase activity mainly involves two enzymes, β-glucosidase [EC:3.2.1.21] and endoglucanase [EC:3.2.1.4]. Following a BlastKOALA search, we identified three endoglucanases [EC:3.2.1.4] (ATSA2_1_02976; ATSA2_1_02978; ATSA2_1_04039) and nine β-glucosidases [EC:3.2.1.21] (ATSA2_1_00441; ATSA2_1_00917; ATSA2_1_01445; ATSA2_1_01993; ATSA2_1_02191; ATSA2_1_02311; ATSA2_1_03061; ATSA2_1_03417; ATSA2_1_03813) in the ATSA2^T^ genome (Additional file 2: Table S1).

##### Chitinase

Some functional genes related to chitinase production, such as chitodextrinase precursor [EC:3.2.1.14] and β-hexosaminidase [EC 3.2.1.52], were annotated from the whole genome (Additional file 2: Table S1).

##### Amylase

Neither strain ATSA2^T^ nor the *Saccharibacillus* genus show any amylase activity (Rivas et al. 2008; Darji et al. 2021). Furthermore, the ATSA2^T^ genome only harbored a *glgA* gene (Additional file 2: Table S1), thus potentially providing an explanation for the apparent absence of amylase activity.

##### Biofilm

Biofilm formation is an important stress-induced survival mechanism that aids in the colonization of many bacteria. Genome annotation highlighted many genes linked to biofilm formation in the ATSA2^T^ genome, including *efp* (elongation factor P), the *flg* gene cluster (*flgBCDEFGLKM*), the *mot* gene cluster (*motAB*), and two *hfq* genes (host factor-I protein) (Additional file 2: Table S1).

##### Iron acquisition

Iron, an essential nutrient required for plant growth, is present in the soil but is largely unavailable to plants due to its low bioavailability. Here, we investigated the ability of the ATSA2^T^ strain to synthesize proprietary siderophores. While phenotypic analyses of siderophores were positive, the siderophore biosynthetic gene cluster (*dhbABCDEF)* that was identified in the *Bacillus* strains was not present in the ATSA2^T^ genome. However, many iron-siderophores involved in the regulation of siderophore uptake, including *fhuABD*, *afuABC*, *fbpABC*, and *fepCDG*, and many gene clusters were identified (Table 2). In addition, the most common key regulators, the *Fur* (ferric uptake regulator) family (Kramer et al. 2020; Ellermann and Arthur 2017), and transcriptional repressors of siderophore synthesis were identified. These included *perR* (Fur family transcriptional regulator, peroxide stress response regulator), *fur*, *zur*, and *furB* (Fur family transcriptional regulator, ferric uptake regulator).

##### Phosphorus, nitrogen, sulfur, and ammonia acquisition

Phenotypic analyses revealed that the ATSA2^T^ strain also solubilizes insoluble phosphate to convert it into an available form of phosphate. Bacteria solubilize immobilized phosphate by gluconic acid production, which is facilitated by glucose dehydrogenase (*gdh*) (Sashidhar and Podile 2010). Importantly, we identified *gdh* (glucose 1-dehydrogenase [EC:1.1.1.47]) and *gdhA* (glutamate dehydrogenase (NADP+) [EC:1.4.1.4]) in the ATSA2^T^ genome. Moreover, *phoABHLU* and *phnCDEP* were also identified in the ATSA2^T^ genome, causing bacterial degradation of phosphonate to phosphate (Table 2). In addition, the major transport system in this strain is a phosphate-specific transporter (*pstABCHS*) gene cluster. The presence of these genes present in the ATSA2^T^ genome suggests that this strain is capable of converting inorganic phosphate into a soluble form. Moreover, the presence of the *phn* gene cluster also indicates that this strain can promote increased phosphate uptake in the plant, thereby aiding plant growth.

As observed in the phenotypic nitrogen fixation data, the ATSA2^T^ strain does not grow on nitrogen-free Jensen's medium, which suggests that it is incapable of nitrogen fixation. Consistent with our phenotypic data, this strain does not contain a nitrogen fixation cluster (e.g., *nif*). However, the *iscU* and *nifU* genes respond to nitrogen fixation, and some gene-related nitrate reductases/nitrite oxidoreductases, including *nar* and *nir* gene clusters for nitrite reductase and nitrite transport, respectively, were annotated in the ATSA2^T^ genome (Additional file 2: Table S1). Other PGP traits, such as sulfate reduction, are listed in Additional file 2: Table S1. Although our phenotypic data did not identify ammonia or sulfate activity, some genes related to ammonia assimilation, including the *cys* gene cluster (*cysCHJH*), were annotated (Additional file 2: Table S1). Some genes associated with ammonia assimilation were also identified in the ATSA2^T^ genome. Functional annotation identified genes responsible for ammonia assimilation via the glutamate dehydrogenase (GDH) pathway by glutamate dehydrogenase-like *gdhA* [glutamate dehydrogenase (NADP+)] and via the glutamine synthetase (GS)-glutamate synthase (GOGAT) pathway using glutamine synthetase-like *glnA* (GLUL; glutamine synthetase) and *glnR* (MerR family transcriptional regulator, glutamine synthetase repressor) (Additional file 2: Table S1).

##### Prediction of useful secondary metabolites

**Fig. 3:**
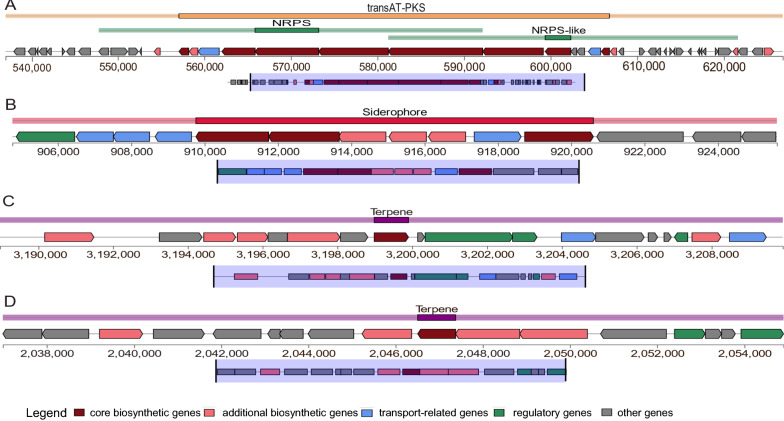
Graphical representation of bacillaene, siderophores (staphylobactin), terpenes, and carotenoids, which are implicated in direct and indirect mechanisms of plant growth

We investigated the strain ATSA2^T^ to understand its effect on plant growth in a greenhouse. We also explored the potential variety of secondary metabolites produced by this strain by using biosynthetic gene cluster (BGC) prediction with antiSMASH. The results highlighted eight notable BGC regions in the ATSA2^T^ genome (Table 3), including bacillaene, staphylobactin, carotenoid, cerecidin/cerecidin A1/cerecidin A2/cerecidin A3/cerecidin A4/cerecidin A5/cerecidin A6/cerecidin A7, and isocomplestatin. Each of these BGCs includes core biosynthetic, regulatory, transport-related, and other genes (Fig. 3A, B). Of the eight known BGCs, four [terpenes, carotenoid, siderophore (staphylobactin), and bacillaene] have been well studied and were shown to be directly or indirectly implicated in PGP (Piccoli and Bottini 2013; Huang and Osbourn 2019; Bottini et al. 2004; Fiodor et al. 2021; Masunaka et al. 2011; Salas-Marina et al. 2011; Yuan et al. 2015; Swapnil et al. 2021; Sulochana et al. 2014; Lurthy et al. 2020; Iqbal et al. 2021b). These four BGCs were found between loci 2,036,992 and 2,054,897 bp (total 17,906 bp) for carotenoid, between 3,188,983 and 3,209,900 bp (20,918 bp) for cerecidin, between 904,761 and 925,605 bp (20,845 bp) for siderophore (staphylobactin), and between 537,059 and 626,796 bp (total 89,738 bp) for bacillaene. However, most of these predicted BGCs showed a low similarity (11–33%) to the known cluster. In addition, one NRPS and one protein did not show any similarity to the known clusters (Table 3). This finding suggested that these BGCs may be new secondary metabolites that require further investigation.

**Table 3:** Predicted secondary metabolite loci in the *S. brassicae* ATSA2^T^ genome

Region	Type	From	To	Most similar known cluster		Similarity (%)
Region 1	TransAT-PKS, NRPS-like, NRPS	1	45,954			
Region 2	TransAT-PKS, NRPS, NRPS-like	537,059	626,796	Bacillaene	Polyketide + NRP	21
Region 3	Siderophore	904,761	925,605	Staphylobactin	Other	18
Region 4	NRPS	1,025,383	1,070,726			
Region 5	Terpene	2,036,992	2,054,897	Carotenoid	Terpene	33
Region 6	Terpene	3,188,983	3,209,900	Cerecidin/cerecidin A1_A7	RiPP: lanthipeptide	11
Region 7	Proteusin	3,917,189	3,937,461			
Region 8	NRPS	5,592,434	5,619,468	Isocomplestatin	NRP	25

*NRPS* nonribosomal peptide synthase, *NRP* nonribosomal peptide, *LAP* lantipeptide, *RiPP* ribosomally synthesized and posttranslationally modified peptides

## Discussion

The genus *Saccharibacillus* belongs to the family *Paenibacillaceae* and is mainly isolated as plant endophytes and from the phyllosphere from plants such as cotton, wheat, vegetables and fruits, sugar cane, and barley seed. Furthermore, this genus can also be found in desert soil and mine tailings (Rivas et al. 2008; Yang et al. 2009; Han et al. 2016; Kampfer et al. 2016; Sun et al. 2016; Besaury and Remond 2020; Jiang et al. 2020; Darji et al. 2021). Plant-associated bacteria, especially plant endophytes, play an important role in PGP and productivity (Santoyo et al. 2016; Hardoim et al. 2008; Singh et al. 2021; Chen et al. 2019). To the best of our knowledge, there have been no studies on interactions between bacteria of the genus *Saccharibacillus* and plants. The genus *Saccharibacillus* has been proven to have strong cellulase activity (Rivas et al. 2008; Darji et al. 2021). Cellulase, a cell wall-degrading enzyme, can affect the structural integrity of the host plants, thereby indirectly promoting host plant growth, suggesting a role for bacteria of the genus *Saccharibacillus* in plant growth and/or biocontrol. This finding is significant in light of the increasing applications being identified for biofertilizers in modern agriculture (e.g., the GRAS *Bacillus* strain) (Chen et al. 2019; Majeed et al. 2018; Bhardwaj et al. 2014; Zaid et al. 2022; Zhao et al. 2022; Thiruvengadam et al. 2022). In the present study, we found that the strain ATSA2^T^, which is an endophyte of kimchi cabbage, contains no antibiotic resistance genes or virulence genes. Therefore, this bacterial strain is likely safe for both humans and the environment.

Endophyte bacteria promote plant growth via the regulation of various plant hormones (e.g., IAA) or increasing nutrient uptake. IAA is a natural auxin that aids bacterial biosynthesis via the l-tryptophan metabolism pathway or via the l-tryptophan independent pathway. IAA is the most common plant auxin and regulates various aspects of plant growth and development. IAA also enhances both cell elongation and cell division and is essential for plant tissue differentiation. More importantly, IAA also induces auxin-dependent lateral root formation, root hair development, and primary root growth, which contribute to PGP. Many bacterial species [e.g., *Pseudomonas* (Singh et al. 2021) and *Enterobacter* (Guo et al. 2020)] promote plant growth via IAA synthesis. Siderophore and phosphate solubilization are also very important to plant growth-promoting traits. Bacteria produce and secrete siderophores for iron absorption, iron transfer into cells, and iron scavenging from the host to inhibit plant pathogens. These mechanisms act indirectly to promote plant growth. Phosphorous is an essential macronutrient for plant growth, but the majority of phosphate is not bioavailable. As such, phosphate-solubilizing bacteria enhance plant production by solubilizing insoluble phosphorus to increase its bioavailability and improve phosphorus nutrition. Some PGP microbes improve plant growth by solubilizing insoluble phosphates in the soil, particularly in phosphorus-deficient environments, thereby increasing phosphorus cycling and improving soil quality. Many bacterial strains, including *Pseudomonas* (Singh et al. 2021; Asaf et al. 2018; Saha et al. 2022; Rikame and Borde 2022; Nishu et al. 2022), *Enterobacter* (Guo et al. 2020; Raturi et al. 2022), and *Serratia* (Adam et al. 2016), promote plant growth by improving nutrient availability. In the present study, we showed that an isolated strain ATSA2^T^ from surface-sterilized seeds produces a relatively high IAA content in the presence of l-tryptophan, with detectable siderophore and phosphate solubilization activity (Table 1). We showed that these mediators promote growth in kimchi cabbage, bok choy, and pepper under greenhouse conditions (Fig. 1, Additional file 1: Fig. S1).

We used whole-genome sequencing and annotation to provide useful insights into the mechanisms governing plant growth. In a previous study, many plant growth-promoting bacteria were analyzed at the whole-genome level to gain an in-depth understanding of PGP mechanisms in bacteria such as *Bacillus pumilus* strain SF-4 (Iqbal et al. 2021a), *B. subtilis* BS87 and *B. megaterium* BM89 (Chandra et al. 2021), *Pseudomonas aeruginosa* B18 (Singh et al. 2021), *Klebsiella variicola* UC4115 (Guerrieri et al. 2021), and *Streptomyces* (Subramaniam et al. 2020). Many genes have been identified by whole-genome sequencing and have been implicated in direct mechanisms of plant growth (e.g., chitinase, phosphate solubilization, auxin production, iron acquisition, and nitrogen fixation) (Iqbal et al. 2021a; Chandra et al. 2021; Singh et al. 2021; Guerrieri et al. 2021; Subramaniam et al. 2020). In the present study, the strain ATSA2^T^ showed high IAA synthesis. In agreement with this observation, the *trpABCDEFG* gene cluster—which is involved in IAA production—was annotated in the ATSA2^T^ genome. The *trp* gene cluster is involved in tryptophan biosynthesis and associated with IAA biosynthesis (Gupta et al. 2014; Singh et al. 2021; Babalola et al. 2021; Asaf et al. 2018; Guo et al. 2020). Furthermore, the occurrence of the following gene clusters, which are responsible for IAA production, also supports our findings: *trpABCDEG*, *trpBCDES*, *trpABCDR*, *trpABD*, and *trpBE* from whole-genome analysis of strains *Pseudomonas aeruginosa* B18 (Singh et al. 2021), *Rhizobacteria* (Gupta et al. 2014), *Bacillus cereus* T4S (Babalola et al. 2021), *Sphingomonas* sp. LK11 (Asaf et al. 2018), and *Enterobacter roggenkampii* ED5 (Guo et al. 2020), respectively. A key gene, *ipdC*, is involved in the IAA biosynthetic pathway and has been identified in *Klebsiella* sp. D5A (Liu et al. 2016). Moreover, the *patB* gene, which constitutes a potential biosynthetic IAA pathway in *B. amyloliquefaciens* Ba13, was also found in the ATSA2^T^ genome (Ji et al. 2021). Importantly, these bacterial strains have been proven to have plant growth-promoting activity. The strain ATSA2^T^ used in this study also showed consistent plant growth-promoting activity in kimchi cabbage, bok choy, and pepper.

Regarding siderophore acquisition, iron is a known essential nutrient that promotes bacterial virulence but must be scavenged by the microbe. Bacteria have several iron transporters (e.g., *Ybt*, *Feo*, *Efe*, *Yfe*, *Fet*, and *Fhu* in *Yersinia pestis*) (Forman et al. 2007). Among those iron transporters, the *Fhu* system was first identified in *Escherichia coli* (Kammler et al. 1993) and participates in siderophore (hydroxamate)-dependent iron (III) transport, with *FhuD* being a siderophore receptor. In the present study, *FhuABD*, which is part of the *Fhu* family, was detected in the ATSA2^T^ genome. Furthermore, similar to our previous study, *FhuBCD* was also identified in *Streptomyces* as a siderophore transport system (Subramaniam et al. 2020). In addition, other genes implicated in the iron-siderophore transport system, including but not limited to *SirABC*, *FecBCD*, *CbrABC*, *FeoB*, *FtsABC*, *SiuABD*, *EfeO*, and *FagABC* (Table 2), were found in the ATSA2^T^ genome. Similar observations were made in *Staphylococcus* (*SirABC*) (Dale et al. 2004), *Shigella flexneri* (*FecIRABCDE*) (Luck et al. 2001), *E. coli* (*FeoAB*) (Kammler et al. 1993), and *Y. pestis* (*Efe*, *Yfe*, and *Fet*) (Forman et al. 2007). Genome analysis revealed that the strain ATSA2^T^ also contains siderophore acquisition activity, which agrees with our phenotypic data and previous findings. We also identified some genes involved in phosphate uptake and solubilization, some of which have been extensively studied, including *phoA* (alkaline phosphatase), *pst* (Pi-specific transporter), *phn* (alkaline phosphatase affinity transport system), and *ugp* (glycerol-3-P uptake) (Martin and Liras 2021; Gebhard et al. 2006). We identified the phosphate solubilization-related gene clusters *phnCDEP*, *phoABHLU*, *pstABCSH*, and *ugpABE* in the ATSA2^T^ genome (Table 2). Furthermore, *pstSCAB*, *phoACX*, and *phnCDE* have all been found in *Streptomyces* and *Mycobacterium tuberculosi*s genomes (Martin and Liras 2021; Gebhard et al. 2006).

Although some extracellular enzyme phenotypes of the strain ATSA2^T^ (e.g., cellulase, amylase, and chitinase activities) were negative, this strain contains genes encoding cellulase (*bglBX*, *ramA*), amylase (*glgA*), and chitinase (*nagZ*) activities, which have been reported in *Streptomyces* and *B. cereus* and *B. subtilis* (Subramaniam et al. 2020; Adeleke et al. 2021; Guo et al. 2015). Similarly, we identified genes involved in nitrogen metabolism in the strain ATSA2^T^, including genes implicated in nitrate transport (*narGIJKQWVHYZI*, *narGZ*, and *nasA)* and nitrite reduction (*nirBCD*) (Additional file 2: Table S1). Interestingly, cellulase activity is strain-dependent, with *S. sacchari* and *S. alkalitolerans* showing significant cellulase isozyme activity (Rivas et al. 2008; Darji et al. 2021), while *S. kuerlensis* showed no cellulase activity (Yang et al. 2009). Our data agree with these previously published findings. In addition to plant-related traits in the present analysis, using whole-genome analysis, we also found that the strain ATSA2^T^ produces volatile compounds via whole-genome analysis. Increasing evidence suggests that volatile compounds produced by bacteria and fungi can stimulate plant growth via processes that are dependent on changes in the metabolome and/or proteome. Furthermore, volatile compounds, including 2,3-butanediol and methanethiol isoprene, have been shown to promote plant growth (Yi et al. 2016; Jardine et al. 2016) in the most efficient plant growth-promoting bacteria, *Bacillus* sp. (Guo et al. 2015; Yi et al. 2016) and *Pseudomonas* sp. In the present study, the 2,3-butanediol-related gene cluster *ilvABCDEGHLN* was annotated from the whole genome, similar to the occurrence of the gene cluster *ilvABCDEH*, *ilvHC* in the whole genome of *Bacillus* sp. and *P. aeruginosa* B18 (Singh et al. 2021; Guo et al. 2015). The methanethiol isoprene-related gene cluster *metABCEGINKQXY* in the genome of ATSA2^T^ was similar to that in *Pseudomonas aeruginosa* B18 (Singh et al. 2021). The production of 3-butanediol and methanethiol isoprene from strain ATSA2^T^ must be further studied.

Plant growth-promoting bacterial strains are often mediated by producing important secondary metabolites that act as a reservoir of bioactive metabolites, such as inhibiting pathogen growth (Kiesewalter et al. 2021). These biocontrol compounds, including fengycin, are produced by *B. subtilis* and *B. velezensis* strains that inhibit *Rhizoctonia* disease and *Aspergillus flavus*, respectively (Deleu et al. 2008; Chen et al. 2019). Iturin A is produced by *B. amyloliquefaciens* and suppresses the biopathogen *Rhizoctonia solani* and PGP (Kushwaha et al. 2020; Murata et al. 2013; D'Aes et al. 2011). Identification and characterization of secondary metabolites is the traditional approach for elucidating the complete chemical structure (Deleu et al. 2008; D'Aes et al. 2011; Blin et al. 2013). This process can be accelerated by using antiSMASH bioinformatic analysis to predict secondary metabolite clusters (Blin et al. 2013) and better characterize the genetic determinants related to plant growth and/or biocontrol activity (Nasrin et al. 2015; Nelson et al. 2014). There are seven published species of the genus *Saccharibacillus*; however, limited secondary metabolites have been studied, and the potential source of natural products in this genus remains untapped. Bacteriocin, thiopeptide, terpene, and nonribosomal peptide (NRP) synthase clusters have been identified in the *S. alkalitolerans* VR-M41^T^ genome (Darji et al. 2021). Among these compounds, some bacteriocin is produced by plant growth-promoting bacteria and can promote plant growth (Gray et al. 2006; Lee et al. 2009). Moreover, thiopeptides and terpenes also have potent antibiotic (Awolope et al. 2021) and plant growth-promoting activities (Abdel-Hamid et al. 2021; Brookbank et al. 2021), which emphasizes the potential plant growth-promoting activity of *Saccharibacillus*.

In the present study, the strain ATSA2^T^ was investigated for biosynthetic secondary metabolites. Eight notable BGC regions were detected. These encode terpenes, siderophores, proteusins, NRPs and NRP-like compounds such as bacillaene, staphylobactin, carotenoids, cerecidin and isocomplestatin. Some of these BGCs were previously unknown. Four BCGs, terpene, carotenoid, siderophore (staphylobactin), and bacillaene, are known to be related to direct or indirect plant growth mechanisms. Terpenes include ABA, GAs, phytoalexins, and membrane-related sterols (Piccoli and Bottini 2013; Huang and Osbourn 2019; Bottini et al. 2004). ABA helps plants maintain their cell turgor to preserve water and indirectly stimulates plant growth, while GAs promote root and shoot growth (Piccoli and Bottini 2013; Fiodor et al. 2021). In addition, phytoalexins can protect plants against pathogens, thereby directly promoting plant growth (Masunaka et al. 2011; Salas-Marina et al. 2011). Carotenoids are a group of isoprenoid metabolites that are vital for diverse plant functions, such as pigmentation and signaling. Increasing evidence shows that carotenoids play an important role in plant growth and improve both plant yield and nutritional value (Yuan et al. 2015; Swapnil et al. 2021). Siderophores are low-molecular-mass compounds that have been shown to promote plant growth via suppression of pathogen growth and by increasing iron from the environment (Sulochana et al. 2014; Lurthy et al. 2020). Bacillaene is a polyene synthesized by trans-acyltransferase polyketide synthases via inhibition of prokaryotic protein biosynthesis. Furthermore, this compound has antibacterial activity, which indirectly promotes plant growth (Ji et al. 2021; Chen et al. 2019; Iqbal et al. 2021b). Additional research on these secondary metabolites is required to fully elucidate the functions of each metabolite in plant growth.

An endophytic bacterium, strain ATSA2^T^, was isolated from seeds of kimchi cabbage (Jiang et al. 2020). We demonstrated tryptophan-dependent IAA production in this strain along with phosphate solubilization and siderophore activity, all of which have contributed to mechanisms of plant growth in kimchi cabbage, bok choy, and pepper in a greenhouse test. Whole-genome sequencing was performed to mine functional genes and IAA-, phosphate solubilization-, and siderophore-related gene clusters. These genes were all identified and highly correlated with our phenotypic data. In addition, secondary metabolites, including carotenoids, siderophores (staphylobactin), and bacillaene, underlining PGP were also identified in the ATSA2^T^ genome by antiSMASH. These data show that genomic analysis offers comprehensive insights into the plant growth-promoting mechanisms of the strain ATSA2^T^, thereby suggesting a role for this bacterial strain in biotechnological applications in agriculture for promoting growth in kimchi cabbage, bok choy, and pepper.

In summary, analysis of the *Saccharibacillus brassicae* ATSA2^T^ genome confirmed its abilities as a PGP through revealing several potential genes involved in plant-growth promotion, such as the biosynthesis of hormone (IAA), siderophore biogenesis, and phosphate solubilization. Our results add important information regarding *Saccharibacillus brassicae* plant growth-promoting abilities and that can inspire further application in sustainable agriculture.

## Supplementary Information

**Additional file 1: Figure S1.** Seed germination and plant growth promotion of rice and Micro-Tom by strain ATSA2^T^. (A, B) Seed germination rate (%) of rice and Micro-Tom with and without ATSA2^T^ inoculation were determined at 3 and 7 days after germination. (C) Effect of strain ATSA2^T^ on rice and Micro-Tom plant growth for 7 and 14 days. Seedlings were inoculated with and without ATSA2^T^ inoculation. Asterisks (*) indicate a significant difference between control (CK) and ATSA2^T^ inoculation (*P < 0.05, *t*-test). **Figure S2.** Effect of the strain ATSA2^T^ on bok choy plant growth. (A) Representative photograph showing the effects of ATSA2^T^. (B) The average leaf number, leaf fresh weight, and root fresh weight of plants by strain ATSA2^T^ treatment. Asterisks (*) indicate a significant difference between control (CK) and ATSA2^T^ inoculation (*P < 0.05, **P < 0.01 and ***P < 0.001, *t*-test).

**Additional file 2: Table S1.** Genes involved in the plant growth-promoting traits based on BlastKOALA and RastSEED.

## Data Availability

The strain ATSA2^T^ is available from the Korea Collection for Type Cultures (KCTC 43072^T^) and the China Center for Type Culture Collection (CCTCC AB 2019223^T^). The GenBank accession number of the strain ATSA2^T^ for the 16S rRNA gene is MN100138. Whole-genome sequences of strain ATSA2^T^ is CP041217. The associated BioSample and BioProject accession numbers are SAMN11812191 and PRJNA544163, respectively. The taxonomy ID is 2583377. All the figures and raw data can be accessed at https://github.com/meng2005/Plant-growth-promoting-bacteria.git.

## Abbreviations

IAA: Indole-3-acetic acid
WGS: Whole-genome sequencing
BGCs: Biosynthetic gene clusters
NRPS: Nonribosomal peptide synthetase
PKS: Polyketide synthase
PGP: Plant growth promotion
NBRIP: National Botanical Research Institute’s phosphate growth medium
LAP: Lantipeptide
RiPP: Ribosomally synthesized and posttranslationally modified peptides
KEGG: Kyoto Encyclopedia of Genes and Genomes
COG: Cluster of orthologous groups
CDSs: Protein-coding genes
CGE: Center for Genomic Epidemiology
CMC: Carboxymethylcellulose
