# Supplementary material for: Genome insights into the plant growth-promoting bacterium Saccharibacillus brassicae ATSA2T
Source: AMB Express. 2023 Jan 21;13:9. doi: 10.1186/s13568-023-01514-1 (PMC9867790; doi:10.1186/s13568-023-01514-1)
Supplement: Supplementary file 1 — Additional file 1: Figure S1. Seed germination and plant growth promotion of rice and Micro-Tom by strain ATSA2T. (A, B) Seed germination rate (%) of rice and Micro-Tom with and without ATSA2T inoculation were determined at 3 and 7 days after germination. (C) Effect of strain ATSA2T on rice and Micro-Tom plant growth for 7 and 14 days. Seedlings were inoculated with and without ATSA2T inoculation. Asterisks (*) indicate a significant difference between control (CK) and ATSA2T inoculation (*P < 0.05, t-test). Figure S2. Effect of the strain ATSA2T on bok choy plant growth. (A) Representative photograph showing the effects of ATSA2T. (B) The average leaf number, leaf fresh weight, and root fresh weight of plants by strain ATSA2T treatment. Asterisks (*) indicate a significant difference between control (CK) and ATSA2T inoculation (*P < 0.05, **P < 0.01 and ***P < 0.001, t-test). [file 13568_2023_1514_MOESM1_ESM.pdf]

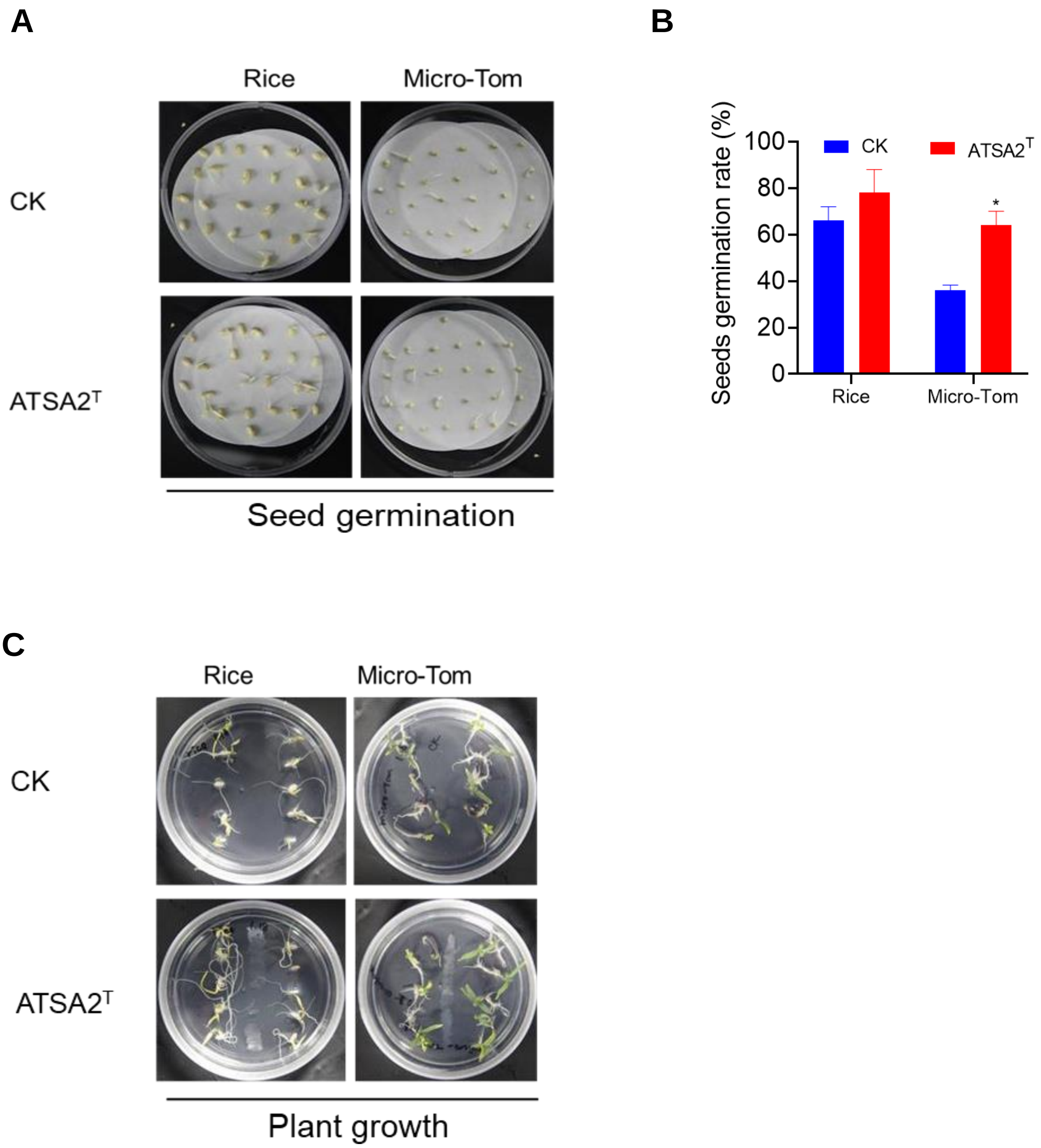

Figure S1. Seed germination and plant growth promotion of rice and Micro-Tom by strain ATSA2^T. (A-B) Seed germination rate (%) of rice and Micro-Tom with and without ATSA2^T inoculation were determined at 3 and 7 days after germination. (C) Effect of strain ATSA2^T on rice and Micro-Tom plant growth for 7 and 14 days. Seedlings were inoculated with and without ATSA2^T inoculation. Asterisks (*) indicate a significant difference between control (CK) and ATSA2^T inoculation (*P<0.05, *t*-test).

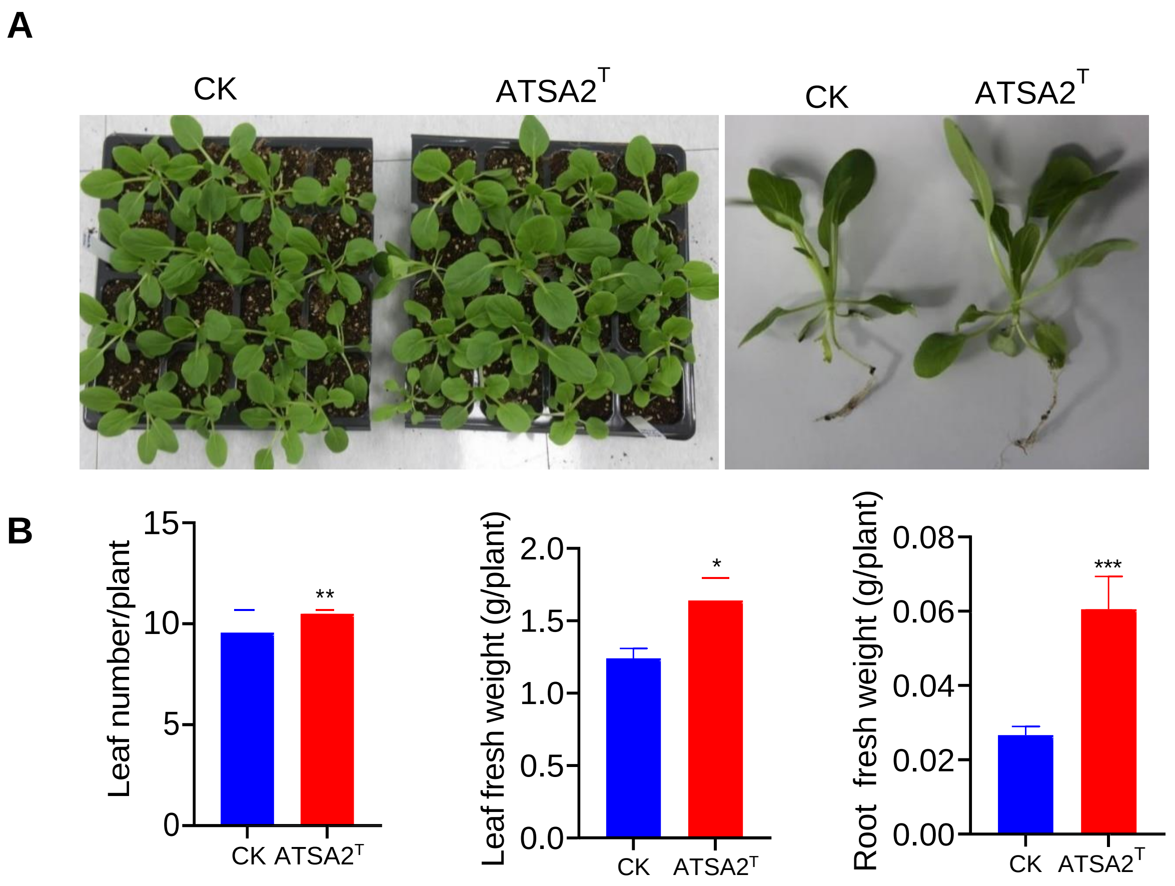

Figure S2. Effect of the strain ATSA2^T on bok choy plant growth. (A) Representative photograph showing the effects of ATSA2^T. (B) The average leaf number, leaf fresh weight, and root fresh weight of plants by strain ATSA2^T treatment. Asterisks (*) indicate a significant difference between control (CK) and ATSA2^T inoculation (*P<0.05, **P<0.01 and ***P<0.001, t-test).
