# Supplementary material for: Genome insights into the plant growth-promoting bacterium Saccharibacillus brassicae ATSA2T
Source: AMB Express. 2023 Jan 21;13:9. doi: 10.1186/s13568-023-01514-1 (PMC9867790; doi:10.1186/s13568-023-01514-1)
Supplement: Supplementary file 2 — Additional file 2: Table S1. Genes involved in the plant growth-promoting traits based on BlastKOALA and RastSEED. [file 13568_2023_1514_MOESM2_ESM.pdf]

Table S1. Genes involved in the plant growth-promoting traits based on BlastKOALA and RastSEED.

Trait	Gene	Gene Annotation	E.C. Number
Cellulase	*bglB*	Beta-glucosidase	[EC:3.2.1.21]
	bglX	Beta-glucosidase	[EC:3.2.1.21]
	ramA	Endoglucanase	[EC:3.2.1.4]
Chitinase	*ND*	Chitinase	[EC:3.2.1.14]
	HEXA_B	Hexosaminidase	[EC:3.2.1.52]
	nagZ	Beta-N-acetylhexosaminidase	[EC:3.2.1.52]
Amylase	*glgA*	Starch synthase	[EC:2.4.1.21]
2,3-Butanediol	*ilvD*	Dihydroxy-acid dehydratase	[EC:4.2.1.9]
	ilvE	Branched-chain amino acid aminotransferase	[EC:2.6.1.42]
	ilvA, tdcB	Threonine dehydratase	[EC:4.3.1.19]
	ilvB, ilvG, ilvI	Acetolactate synthase I/II/III large subunit	[EC:2.2.1.6]
	ilvH, ilvN	Acetolactate synthase I/III small subunit	[EC:2.2.1.6]
	ilvC	Ketol-acid reductoisomerase	[EC:1.1.1.86]
	isp	Major intracellular serine protease	[EC:3.4.21.-]
	gcpE, ispG	(E)-4-hydroxy-3-methylbut-2-enyl-diphosphate synthase	[EC:1.17.7.1 1.17.7.3]
	ispD	2-C-methyl-D-erythritol 4-phosphate cytidylyltransferase	[EC:2.7.7.60]
	ispE	4-diphosphocytidyl-2-C-methyl-D-erythritol kinase	[EC:2.7.1.148]
	ispH, lytB	4-hydroxy-3-methylbut-2-en-1-yl diphosphate reductase	[EC:1.17.7.4]
Methanethiol isoprene	*metA*	Homoserine O-succinyltransferase/O-acetyltransferase	[EC:2.3.1.46 2.3.1.31]
	metB	Cystathionine gamma-synthase	[EC:2.5.1.48]
	metC	Cysteine-S-conjugate beta-lyase	[EC:4.4.1.13]
	metG	Methionyl-tRNA synthetase	[EC:6.1.1.10]
	metE	5-methyltetrahydropteroyltriglutamate--homocysteine Methyltransferase	[EC:2.1.1.14]
	metQ	D-methionine transport system substrate-binding	
	metI	D-methionine transport system permease	
	metN	D-methionine transport system ATP-binding	
	metY	O-acetylhomoserine (thiol)-lyase	[EC:2.5.1.49]
	metK, MAT	S-adenosylmethionine synthetase	[EC:2.5.1.6]
	metX	Homoserine O-acetyltransferase/O-succinyltransferase	[EC:2.3.1.31 2.3.1.46]
Biofilm formation	*efp*	Elongation factor P	
	flgL	Flagellar hook-associated protein 3 FlgL	
	flgK	Flagellar hook-associated protein 1	
	flgM	Negative regulator of flagellin synthesis FlgM	
	flgG	Flagellar basal-body rod protein FlgG	

	flgF	Flagellar basal-body rod protein FlgF	
	flgB	Flagellar basal-body rod protein FlgB	
	flgC	Flagellar basal-body rod protein FlgC	
	flgD	Flagellar basal-body rod modification protein FlgD	
	flgE	Flagellar hook protein FlgE	
	motB	Chemotaxis protein MotB	
	motA	Chemotaxis protein MotA	
	hfq	Host factor-I protein	
Nitrogen fixation	*nirB*	Nitrite reductase (NADH) large subunit	[EC:1.7.1.15]
	iscU, nifU	Nitrogen fixation protein NifU and related proteins	
	narG, narZ, nxrA	Nitrate reductase / nitrite oxidoreductase, alpha subunit	[EC:1.7.5.1 1.7.99.-]
	narH, narY, nxrB	Nitrate reductase / nitrite oxidoreductase, beta subunit	[EC:1.7.5.1 1.7.99.-]
	narI, narV	Nitrate reductase gamma subunit	[EC:1.7.5.1 1.7.99.-]
	narJ, narW	Nitrate reductase molybdenum cofactor assembly Chaperone NarJ/NarW	
	narQ	Two-component system, NarL family, nitrate/nitrite sensor Histidine kinase NarQ	[EC:2.7.13.3]
	nasA	Assimilatory nitrate reductase catalytic subunit	[EC:1.7.99.-]
	nirB	Nitrite reductase (NADH) large subunit	[EC:1.7.1.15]
	nirC	Nitrite transporter	
	nirD	Nitrite reductase (NADH) small subunit	[EC:1.7.1.15]
	nreA	Nitrogen regulatory protein A	
	NRT, narK, nrtP, nasA	MFS transporter, NNP family, nitrate/nitrite transporter	
	iscS	NFS1; cysteine desulfurase	[EC:2.8.1.7]
	iscA	Iron-sulfur cluster assembly protein	
	gltD	Glutamate synthase (NADPH) small chain	[EC:1.4.1.13]
	gltX	Nondiscriminating glutamyl-tRNA synthetase	[EC:6.1.1.24]
	gltB	Glutamate synthase (NADPH) large chain	[EC:1.4.1.13]
	gltA	Citrate synthase	[EC:2.3.3.1]
	gltX	Glutamyl-tRNA synthetase	[EC:6.1.1.17]
	yeiL	CRP/FNR family transcriptional regulator, Putative post-exponential-phase nitrogen-starvation regulator	
Potassium solubilization	*kdpA*	Potassium-transporting ATPase potassium-binding subunit	
	kch, trkA, mthK, pch	Voltage-gated potassium channel	
	kdpB	Potassium-transporting ATPase ATP-binding subunit	[EC:7.2.2.6]
	kdpC	Potassium-transporting ATPase KdpC subunit	
	trkA, ktrA, ktrC	Trk/ktr system potassium uptake protein	

trkH, trkG, ktrB, ktrD	Trk/ktr system potassium uptake protein
